# The FCTC dilemma on heated tobacco products

**DOI:** 10.1186/s12992-020-00596-x

**Published:** 2020-09-11

**Authors:** Lukasz Gruszczynski, Margherita Melillo

**Affiliations:** 1 Kozminski University, College of Law, Jagiellonska St. 57/59, 03-301 Warsaw, Poland; 2 Institute for Legal Studies of the Centre for Social Sciences , Tóth Kálmán St. 4, Budapest, H-1097 Hungary; 3 Law Department, European University Institute, Via Bolognese 156, 50139 Florence, Italy; 4 Max Planck Institute Luxembourg for International, European and Regulatory Procedural Law, rue Alphonse Weicker 4, L-2721 Luxembourg, Luxembourg

**Keywords:** Heated tobacco products, HTP, Framework convention on tobacco control, FCTC, Tobacco, Tobacco control, Harm reduction

## Abstract

**Background:**

In October 2018, the Conference of the Parties of the Framework Convention on Tobacco Control (FCTC or Convention) adopted its first decision on novel and emerging tobacco products, including heated tobacco products (HTPs). The decision remains ambiguous, e.g. by making a distinction between tobacco sticks and HTP devices. Against this background, the article seeks to answer two interrelated questions: whether and to what extent HTPs are covered by the FCTC, and whether regime provided by the Convention is suitable for their regulation.

**Results:**

HTPs need to be classified under the FCTC as tobacco products. The distinction made by the Conference of the Parties between sticks and devices leads however to unsatisfactory results as it creates loopholes in tobacco control standards existing at the international level. A better approach, as argued in this article, is to conceptualize the notion of ‘tobacco products’ in functional terms as a combination of both a device and stick.

While subjecting HTPs to all FCTC disciplines is, in light of our current scientific knowledge, a rational approach, such classification can be modified in the future once a sufficient amount of new evidence on their risk profile is collected. Any decision on the optimal regulatory model for HTPs will need to take into account not only health risks and potential benefits for individual users, but also the specific systemic concerns (e.g. HTPs as a gateway product). The state of scientific research is however not the only factor that will determine the fate of HTPs under the Convention. What is equally important is a conceptualization of the FCTC’s objectives. If a complete eradication of the tobacco epidemic is the ultimate goal, reduced levels of risk may not be enough to justify the different (i.e. more lenient) regulatory regime for HTPs.

**Conclusions:**

The Conference of the Parties should clarify the definition of tobacco products in light of recent changes in the market. When designing the regulatory regime for HTPs under the FCTC in the future, one has to consider not only scientific evidence but also pay attention to the objective of the Convention (or more generally to the values that underlie the current tobacco control paradigm).

## Background

The last decade has witnessed some profound changes in the global market for tobacco products. As a consequence of the intensified and coordinated efforts of governments, stimulated by, among other things, the adoption in 2003 of the Framework Convention on Tobacco Control (FCTC or the Convention) [1], many countries have seen a steady decrease in the prevalence of tobacco use. Some states have even announced that they would become smoke-free nations in the relatively near future, meaning that less than 5% of their population will be using tobacco products [2]. Although it remains unclear whether this objective will be actually achieved, many experts believe this could indeed be the final phase of the famous end game [3].

The FCTC is an evidence-based treaty that establishes certain global standards for national tobacco control policies under the auspices of the World Health Organization (WHO). The Convention is almost universally accepted, with 181 Parties that represent about 90% of the global population [4]. The objective of the FCTC is described in its Art. 3, according to which the Convention seeks to ‘protect present and future generations from the devastating health, social, environmental and economic consequences of tobacco consumption and exposure to tobacco smoke.’ To this end the FCTC sets specific tobacco control standards for its Parties, covering both demand- and supply-side aspects (e.g. regulation of the contents of tobacco products; packaging and labelling of tobacco products), as well as other related matters (e.g. the requirement to protect domestic health policies from commercial and other vested interests of the tobacco industry). The Convention also confirms that it only sets minimum standards and does not prevent the Parties from introducing stricter tobacco control measures if they are consistent with its other provisions (Art. 2.1).

Most of the FCTC obligations are formulated in very general language, leaving the Parties with only limited guidance as to their proper implementation. In practice, various provisions are further clarified through the more technical guidelines which are adopted from time to time by the Conference of the Parties (COP) [5]. Although technically speaking guidelines are not legally binding, in practice they are followed by the Parties. The COP may also adopt protocols, annexes, and amendments to the Convention, which however become binding only after the conclusion of national ratification, acceptance, or an approval process (and only for those Parties that have completed such a procedure). Finally, the COP is expected to keep the implementation of the Convention under regular review and take the decisions necessary to promote its effective implementation (Art. 23.5). Those decisions cannot however add to or diminish the existing FCTC obligations.

The Convention is widely seen as one of the major achievements in the field of global public health protection. Its adoption was clearly a catalyst for actions at the national level. Many low- and middle-income countries have introduced effective tobacco control policies for the first time in their history (mostly by copying the requirements provided by the FCTC), while developed states have strengthened their pre-existing standards. These developments have been translated into reductions in smoking prevalence in many countries, particularly those that have a high implementation record [6]. The Convention has also proved to play an important role in legal defenses against the tobacco industry, both at the national and international levels [7], and helped to keep tobacco control at the top of the global political agenda. As correctly summarized in the 2016 report of by the FCTC Expert Group, ‘[w] hile it will never be possible to identify precisely how many measures are directly or indirectly attributable to the Convention, … the FCTC has undoubtedly played a critical role as an authoritative and agreed catalyst and framework for action.’ [8]

However, and at least partially in response to those developments [9], new devices, such as electronic nicotine delivery systems (ENDS or e-cigarettes) or heated tobacco products (HTPs) have appeared on the market, becoming increasingly popular among consumers [10]. ENDS are battery-powered appliances designed to deliver nicotine, but instead of using tobacco they heat a special solution (a so-called ‘e-liquid’), composed of vegetable glycerine, propylene glycol and nicotine, the vapour of which is inhaled. In most of the countries, the markets for ENDS still remain fragmented, with many independent producers (with one notable exception being the United States, where JUUL, as a leading brand, still controls the majority of the traditional retail sales [11]). At the same time, one may also observe progressive consolidation of the sector which is increasingly controlled by the Big Tobacco.

On the other hand, HTPs is a generic name for various devices that use an electric heating element to char, usually at the level of 240–350 °C (but there are models, such as Ploom S, which work at lower temperatures) the processed tobacco in the form of a special stick to be inserted into the electronic device, generating in this process an aerosol that contains nicotine (as well as other chemical substances), and which is inhaled by a user. HTPs began to be commercialized on wider scale in 2014, although some early models were already available at the end of 1980s (e.g. Premier and later Eclipse). The market has been dominated by the largest tobacco companies from its inception, and currently the three leading products are IQOS (owned by Philip Morris International (PMI)), Glo (owned by British American Tobacco) and Ploom (owned by Japan Tobacco International) [12]. In this context, it should be also noted that the name of the product may actually be misleading. First, it does not seem to be entirely neutral as it appeals to the consumers emotions by using an adjective that is normally associated with pleasant feelings. Second, and probably more importantly, it does not necessarily describe what is may actually be happening inside of most HTP devices. Some researchers claim that the temperature generated by a heating element is sufficiently high to cause pyrolysis and thermogenic degradation, the very same physical processes that occur in the course of regular combustion [13]. Consequently, the more precise term to describe this category might be ‘scorched tobacco products’. Nevertheless, since HTPs is a phrase used in the literature, while the evidence on pyrolysis and thermogenic degradation is not conclusive, we follow the existing convention.

Despite important differences, the common element between those two categories of products is that they are both marketed (explicitly or implicitly) as harm reduction devices. According to their advocates, it is the lack of combustion that makes them different from traditional tobacco products, as this affects the level of exposure to the harmful constituents present in tobacco smoke (note that in the context of ENDS lack of tobacco leaf in a final product is also highlighted). However, there are still many uncertainties surrounding the long-term and systemic risks that these products could pose [14]. Could exposure to some of their chemicals be hazardous over the long-term? Could they pose any systemic risks, such as the renormalization of smoking or perpetuation of nicotine addiction?

As far as HTPs are concerned, some studies indeed suggest that their use may pose lower health risks as compared to combustible tobacco [15]. For example, a group of eminent scholars has recently noted in their comprehensive literature review that while all studies indicate that HTPs expose users and bystanders to toxicants, at least some of them show that the levels of specific toxicants are substantially lower than in the case of combustible cigarettes [16]. Similarly, the US Food and Drug Administration (FDA), in its recent premarket decision for IQOS, came to the conclusion that carbon monoxide and formaldehyde exposure was lower as compared to combustible cigarettes [17]. On the other hand, it is also true that the available data remain limited (a fact openly admitted by FDA) and are predominantly based on laboratory, rather than population-based, studies. Consequently, they do not necessarily allow for making any firm determinations as to the safety of those products. In addition, a number of other studies have demonstrated that at least some levels may be actually higher than initially anticipated. For example, as noted by the European Respiratory Society ‘[t] wenty two harmful or potentially harmful substances were >200% higher and seven were >1000% higher than in reference to cigarette smoke … there is no statistically detectable difference between users of heated tobacco and conventional cigarettes for 23 of the 24 biomarkers of potential harm’. [18] Similarly, the WHO states on its official webpage that ‘some tobacco industry-funded studies have claimed that there are significant reductions [for HTPs] in the formation of and exposure to harmful and potentially harmful constituents relative to standard cigarettes. However, there is currently no evidence to suggest that reduced exposure to these chemicals translates to reduced risk in humans.’ [19] In this context, it is also worth noting that the FDA decision has been criticized by a number of leading public health experts, who have claimed that some evidence relating to the risks posed by HTPs was either disregarded or insufficiently appreciated [20]. Overall, the experts seem to agree that HTPs do not guarantee 90–95% reduction in harmful and potentially harmful substances and toxicity (taken as a whole), as sometimes claimed by the industry [21]. Nor is it clear what are the long-term effects of their use, which is a consequence of the relatively short history of these products on the market (and owing to the lengthy lag time for the onset of most of the smoking-related diseases, such as cancer or emphysema). There is also no decisive evidence that would demonstrate whether HTPs can actually help current smokers to quit traditional smoking. An additional layer of difficulty is added by the fact that most of the available studies comes from industry-funded projects. Considering the long and consistent history of the industry’s attempts to manipulate the scientific data [22], one may be legitimately sceptical about the credibility of at least some of its results.

The potential challenges posed by HTPs are, however, not only restricted to the level of an invidual user but may also have a systemic character. For example, it has been suggested that HTPs can act as a gateway product, luring non-smokers into addiction to nicotine (which is particularly problematic in case of adolescents) and thus prolonging, rather than fighting, the tobacco epidemic [23]. Eventually some of those people may either switch to traditional tobacco products or remain permanent or occasional users of HTPs, while in a base-case scenario some of them will never become addicted to nicotine. Although the available evidence is too limited to come up with any definitive conclusions in this regard, it is worth mentioning that one of the surveys organized in Italy in 2018 found that almost half of those who experimented with HTPs (i.e. the IQOS model) had never smoked any traditional tobacco products [24]. Having said this, it should also be added FDA came to the opposite conclusion and indicated that the population most likely to use HTPs (i.e. IQOS) were current smokers rather than non-smokers [25].

Some experts also worry that the availability of these products may undermine delegitimizing strategies implemented over last decades by the States (‘delegitimization’ may be understood as referring to various programs and actions that are ‘undertaken to reinforce the fact that tobacco use is not a mainstream or normal activity in [the] society’ [26]). The emergence of new products, such as HTPs, that are attractively designed and appeal to consumers may actually lead to a re-normalization of tobacco use in the society [27]. Although there is very little evidence that would allow for verifying this hypothesis, considering the values at stake one should not dismiss it lightly. Finally, re-normalization may also extend beyond the mere public perception of smoking and can affect the general position occupied by the tobacco industry in the society (note that the industry has been significantly eradicated from various social processes in many States). In particular this may lead to the recognition of the industry as one of the legitimate actors that actively participates in the public efforts to address specific health risks (i.e. through the supply of reduced-risk products). Indeed, different strategies that have already been implemented by Big Tobacco seem to confirm these concerns. For example, PMI, which in its communications consistently refers to the idea of a smoke-free future, declares that: ‘We understand the millions of men and women who smoke cigarettes. They are looking for less harmful, yet satisfying, alternatives to smoking. We will give them that choice’, and adds that ‘Society expects us to act responsibly. And we are doing just that by designing a smoke-free future’. [28] Such developments will obviously undermine the decades of the efforts of the health communities and may be nothing more than just a strategy to regain influence over the future direction of tobacco control policies [29].

Considering the above, it is not surprising that the emergence of these products has created a serious dilemma for the international health community. Should they be simply regarded as a modern incarnation of tobacco products and an element in the adjustment strategy implemented by the shrinking tobacco industry? Or are they perhaps a useful element of a well-designed harm reduction strategy, allowing some of the current smokers to switch to less harmful alternatives? This dilemma is also relevant for the FCTC, which is the central point of reference for most national health regulators in the area of tobacco control.

This bring us to the core issues of this article. Its main objective is to analyse whether, and to what extent, HTPs are covered by the FCTC obligations. In this context, this article argues that the COP’s approach to HTP devices remains unsatisfactory as it potentially allows these items to escape from the reach of some of the FCTC provisions. This enquiry is supplemented by the related but distinct question of whether the obligations provided by the FCTC are actually suitable in the long-term perspective for regulating such products. The scope of this text is purposefully limited to HTPs and does not cover ENDS. Such an approach seems to be justified by the physical differences between those two categories of the products, which may warrant their different legal treatment. Moreover, the problem of the applicability and suitability of the FCTC rules to ENDS, in contrast to HTPs, has already been addressed in the literature [30]. This article, rather than repeating the relevant findings, intends to fill in the existing research gap.

## Methods

The article is based on the standard research methods used in the area of legal studies [31]. In particular, it examines the text of the FCTC and the relevant decisions of the COP, using the formal-dogmatic (textual) method, in order to identify their normative content. It also uses the teleological and systemic interpretation in analyzing the relevant rules (i.e. interpreting individual legal provisions light of the purpose and goals they aim to achieve as well as considering them in the broader legal context provided by the whole Convention). This analysis is supplemented by the examination of the COP practice. Such an approach closely corresponds with the general rules on interpretation of public international law as set out in Art. 31 of the Vienna Convention on the Law of Treaties (VCLT) [32], the provision which is conventionally seen as reflecting the customary rules of interpretation applicable to all international agreements. It particularly provides that ‘[a] treaty shall be interpreted in good faith in accordance with the ordinary meaning to be given to the terms of the treaty in their context and in the light of its object and purpose.’ The provision also requires to ‘[take] into account, together with the context: (a) any subsequent agreement between the parties regarding the interpretation of the treaty or the application of its provisions; (b) any subsequent practice in the application of the treaty which establishes the agreement of the parties regarding its interpretation; (c) any relevant rules of international law applicable in the relations between the parties’ (para. 3).

On the basis of the above assessment, the article proposes possible modifications to the legal definition of ‘tobacco products’ that would address the detected deficiencies in the application of the FCTC provisions to HTPs. The article also identifies three possible future scenarios and preliminary maps, as *de lege ferenda* postulates, the approaches available to Parties when regulating HTPs as the relevant science develops.

The discussion presented in this article is structured as follows: The first part of the subsequent section (‘Results’) looks at the issue of the applicability of the Convention to HTPs, while the second one examines the reach of the FCTC obligations. In the context of the second part, the article particularly enquires whether HTP devices are also covered by the Convention. The third part moves on to the normative question of the desirability of subjecting HTPs to the FCTC rules. The final section (‘Discussion and conclusions’) considers the implications of the findings in context of existing research and offers the main conclusions.

## Results

### The problem of the FCTC’s applicability to HTPs

The modern debates on policies and regulations in the field of tobacco control have mainly focused on cigarettes, an invention of the late nineteenth century [33]. As such, cigarettes have become a symbol of the power of the tobacco industry. Tobacco, nonetheless, has a longer and more varied history of different modes of consumption, which includes products such as chewing tobacco, snus, cigars, pipes, and waterpipes.

Although the negotiations which led to the conclusion of the FCTC were conceived as a response to the epidemic caused by cigarettes, the FCTC was always meant to cover a broader array of tobacco products. For example, the first draft considered by the Intergovernmental Negotiating Body (i.e. a forum for the inter-state negotiations that drafted the FCTC text) included a long definition of ‘tobacco products’, which listed 14 different types of products [34]. To avoid the risk of having a too narrow or incomplete list, the negotiating Parties eventually decided to provide only a general definition of tobacco products. The FCTC text, hence, now defines tobacco products as ‘products entirely or partly made of the leaf tobacco as raw material which are manufactured to be used for smoking, sucking, chewing or snuffing’ (Art. 1(f)).

At the same time, it is not entirely clear whether the text of the FCTC was meant to cover future products. Most probably, the question was not addressed during the FCTC negotiations. At that time (2000–2003), no new tobacco products had been recently introduced, and there was no indication that new products could or would soon be marketed. However, question as to the scope of the FCTC began arising in 2010, when ENDS became mass market products [35]. The same question on the scope of the FCTC is now relevant to HTPs, the second most widespread product developed after the conclusion of the FCTC. So does the Convention apply to HTPs, and if this is a case, to what extent?

Unlike ENDS, HTPs do contain tobacco, and hence can be regarded as ‘partly made of the leaf of tobacco as raw material’ (the condition which may be problematic for ENDS [36]). On the other hand, it may be disputable whether HTPs meet the second and cumulative condition of the FCTC definition, i.e. whether they are ‘manufactured to be used for smoking, sucking, chewing or snuffing’. The tobacco industry consistently highlights that the emissions produced by HTPs should not be regarded as smoke, but rather as a vapor [37]. In this narrative smoking is understood as a practice whereby a substance is burned, and the resulting smoke is breathed in. Since HTPs heat processed tobacco instead of combusting it, they do not produce any smoke. Recall, however, that the heating process in HTPs may actually lead to pyrolysis and thermogenic degradation, while ‘vapor’ contains carbon monoxide and tar (although at the levels lower than those found in cigarette smoke). Consequently, many researchers persuasively argue that such a vapor should be actually classified as a smoke [38]. Moreover, and from legal point more importantly, the term ‘smoking’ as used in the FCTC can be also read broadly (and still remains within the existing textual boundaries). For example, the Merriam-Webster dictionary defines noun ‘smoke’ not only as ‘the gaseous products of burning materials especially of organic origin made visible by the presence of small particles of carbon’, but also more generally as a ‘fume or vapor often resulting from the action of heat on moisture’. [39] The relevant verb will therefore describe the act of inhaling such a ‘smoke’. Alternatively, one may also refer here to another present participle which is used the FCTC definition of ‘tobacco product’. According to the Cambridge dictionary ‘sucking’ means ‘pull [ing] in liquid or air through … mouth without using … teeth’ [40]. The activity connected with the use of HTPs seems to fall within the scope of this description. Either way, it may be concluded that the HTPs can be legally classified as ‘tobacco products’ under the FCTC [41].

This conclusion finds support in the existing literature and practice. For example, the WHO in its most recent report on the global tobacco epidemic, when discussing HTPs, notes that ‘these aerosols are inhaled by users during a process of sucking or smoking involving a device’. [42] Lempert and Glantz have also observed that the objective of the FCTC is to protect present and ‘future’ generations [43]. The reference to the future clearly indicates that the FCTC takes a forward-looking perspective and implies that yet-to-be developed products should fall with the scope of its provisions. In a similar vein, the Framework Convention Alliance (FCA, the network of civil society organizations working in tobacco control) has noted that one of the FCTC’s guiding principles, enshrined in Art. 4, is ‘the need to take measures to prevent the initiation, to promote and support cessation, and to decrease the consumption of tobacco products *in any form* [44]. We cannot but agree. It would be paradoxical to have a treaty on tobacco control that does not cover the newest tobacco products. The inclusion of a broader definition of ‘tobacco products’, which does not provide any enumeration of specific items, also strongly suggests that the Parties intended that the Convention retain an open character that allows for inclusion of future products. Last but not least, one also has to born in mind that many consumers often use two or more tobacco products [45]. A common framework of regulation is thus needed.

Although, we have concluded that HTPs should be classified as tobacco products, it is worth mentioning that even if our conclusion were different, some provisions of the FCTC would still remain relevant for this category of products. For example, Art. 5.2(b) calls on the FCTC Parties to ‘adopt and implement effective legislative, executive, administrative and/or other measures ( …) for preventing and reducing tobacco consumption [and] nicotine addiction ( …)’ . [46] Having said this, it should be also noted that the language of that provision leaves the Parties with a very broad discretion (essentially unlimited) as to how to deal with HTPs. In this context, one may argue that it provides an equal basis for a whole spectrum of responses ranging from a total ban (because this is a logical way of preventing and reducing the nicotine addiction resulting from the use of HTPs) to a laissez-fair approach, or even some form of proactive strategy (if HTPs are seen by a particular State as a useful harm reduction tool which would lead to reduction of tobacco consumption, exposure to tobacco smoke, and to a lesser extent nicotine addiction). Such a vague formulation of the provision may suggest, however, that it only has an aspiratory character and in practice is deprived of most of its normative value. Fortunately for us, there is no need to resolve this problem here.

While it is easier to answer the question *whether* HTPs are covered by the FCTC, the thornier question becomes: *To what extent* are HTPs covered by the FCTC? More specifically, are FCTC provisions applicable to all the different components of HTPs? Against this backdrop, the next part reviews the history of how the Conference of the Parties to the FCTC (COP) has approached HTPs, and critically assesses its decisions.

### The COP decision on HTPs

The COP took note of HTPs in 2016, 2 years after the first models began to be marketed in a few selected countries [47]. The initial approach was explorative, as the COP requested the FCTC Secretariat to collaborate with the WHO to ‘monitor and examine market developments and usage of novel and emerging tobacco products, such as “heat-not-burn” tobacco products’. [48] Following up on this request, the WHO included a section on HTPs in its report prepared for the subsequent meeting of the COP in 2018. After reviewing the current trends, the WHO did not recommend any specific action other than collecting data to better evaluate the evolution of the trends [49].

The absence of specific recommendations on HTPs by the WHO may at first glance seem surprising. Was the WHO recommending the COP to stay idle in the face of the tobacco industry’s newest ‘global strategy’? Certainly not. A reading of the WHO’s online factsheet perhaps better clarifies the WHO’s stance: HTPs are ‘tobacco products’, and should be regulated as such, ‘in line with’ the FCTC [50]. The FCA echoed this position in its own policy briefing in preparation for the COP: HTPs are tobacco products, and ‘[n] o decision by the COP is required to ensure the relevant [FCTC] articles apply’ [51]. The problem of the applicability of FCTC provisions to HTPs was, however, not as clear-cut as the WHO and the FCA seemed to suggest. In Italy, for example, the tobacco sticks designed for HTPs enjoy a more favourable taxation and fewer regulatory restrictions than other tobacco products such as roll-your-own tobacco or traditional cigarettes [52], arguably because of their harm reduction potential (this is also true for the other European Union (EU) countries) [53]. It is probably for these reasons that, despite the views of the WHO and of the FCA, the EU decided to take action on the matter, and submitted a draft decision on HTPs at the COP [54].

The operative part of the draft decision focused on requesting the FCTC and WHO Secretariats to prepare a comprehensive report on the available evidence concerning HTPs [55]. However, the draft also contained language on the applicability of the FCTC to HTPs. This was judged as insufficient by many Parties, since it only ‘recommended’ – not ‘requested’ – the Parties to apply the FCTC tobacco control measures to HTPs [56]. Arguably this could have been regarded by (at least some of) the Parties as a step backwards in the implementation of the FCTC obligations.

Following long discussions in an informal drafting group [57], the EU’s text came out substantially revised. The final version was adopted by the COP as a decision on ‘novel and emerging tobacco products’ [58]. In addition to requesting the WHO and FCTC Secretariats to provide more detailed information on HTPs, the decision ‘remind [ed] Parties about their commitments under the WHO FCTC when addressing the challenges posed by novel and emerging tobacco products’ [59]. As a part of the reminder, the decision also asked the Parties to consider ‘prioritizing’ certain FCTC measures, such as smoke free-legislation (Art. 8 of the FCTC), measures regarding advertising, promotion and sponsorship (Art. 13 of the FCTC), protection from commercial and other vested interests of the tobacco industry (Art. 5.3 of the FCTC), regulation and disclosure of contents (Arts. 9 and 10 of the FCTC) [60], ‘to prevent the initiation’, ‘to prevent health claims’, and ‘to regulate, including restrict, or prohibit, as appropriate, the manufacture, importation, distribution, presentation, sale and use of novel and emerging tobacco products, … taking into account a high level of protection for human health’. [61] Finally, the decision recommended (i.e. again as a part of the ‘consider prioritizing’ phrase) to ‘apply, *where appropriate*, the above measures to the devices designed for consuming such products’. [62]

### What is missing in the COP decision?

As shown above, within 4 years from the introduction of the first HTP models in a few selected countries (2014), the COP was able to find a consensus to adopt a decision on HTPs (2018). Four years can be considered a relatively short time for the COP to react – especially if compared to the lengthy and rather unfruitful debates that the COP has had on ENDS over the last 10 years [63].

However, whether this rapidity was paralleled by quality of action is a different question. The COP decision was seemingly adopted in a rush. The COP’s initial agenda did not foresee the adoption of a decision on HTPs, but only consideration of the issue. However, after the EU introduced its draft decision, the Parties that considered its text to be too weak made their best efforts to improve it. The FCA commented on these developments in one of its daily bulletins, warning against rushing to adopt a bad draft. ‘On some issues, silence is golden’, they wrote [64]. In other words, it would be better not to have a decision on HTPs at all, than have a *bad* decision. However, the COP Parties adopted a different line of reasoning: better a *mediocre* decision than a *bad* decision. The fear, in all likelihood, was that if they did not do anything at all, the EU’s bad draft would be adopted by the COP.

Accordingly, the decision on HTPs was adopted without having a comprehensive report as a reference point, and without the possibility of seeking more detailed recommendations from experts (including lawyers). Specifically, the decision missed the opportunity to address the one of the most problematic regulatory aspects of HTPs: the regulation of electronic devices that are used to heat the tobacco sticks. As regards HTPs, this device is the electronic component in the shape of ‘fat’ cigarettes (IQOS) or cuboid gadgets (Glo or Ploom S). The device is the most visible and sizeable part of HTPs. It can be sold together with changeable sticks or separately. In the first situation, and as discussed above, it will be easily classified as a tobacco product because of accompanying sticks, however it will lose that status in the second situation, inasmuch as the HTP device does not contain any tobacco. It is apparent, nonetheless, that a device is the essential component of HTPs: without it, tobacco cannot not be heated and vaporised. At the same time, in the absence of the tobacco component, HTP device is ostensibly useless. For this reason, the HTP devices and their tobacco component ought to be simply considered an ‘integrated tobacco product’, irrespectively of their modes of sale [65]. Unfortunately, so far this has not been the case.

There is some evidence that the tobacco industry markets HTP devices separately from the tobacco they use, thereby trying to circumvent existing tobacco control regulations [66]. By selling the electronic device as a discrete product, the tobacco industry is able to advertise and promote its new product, placing posters in points of sale in strategic locations. Moreover, the devices have a minimalistic and attractive design, and are sold in equally minimalistic and attractive stores – possibly taking inspiration from Apple’s marketing strategies [67]. HTP devices, therefore, are where the proverbial devil hides.

Despite these well-known facts, no specific paragraph on HTP devices was included in the initial EU draft submitted at the COP. Over the course of the informal negotiations, it has been reported that ‘new wording was added to indicate that the decision applied both to the products themselves and to any devices required to consume them’. [68] However, the final text of the COP decision is still unsatisfactory, as it only reminds the Parties ‘to apply, where appropriate, the above measures to the devices designed for consuming such products’. [69] In our opinion, this soft and generic wording only provides the Parties with limited assistance, and while it does not prevent them from regulating devices as they wish, neither does it give any clear guidance as to how to deal with this category of products. For example, the expression ‘consider prioritizing’ indicates rather weak recommendation, while ‘as appropriate’ suggests that only some provisions of the FCTC are applicable (but which of them?). In a similar vein, the decision also seems to distinguish between devices and novel tobacco products in a strict sense (‘devices designed for consuming *such products*’ [emphasis added]), which leads to the difficult conceptual questions. If devices as such are not considered to be ‘novel and emerging tobacco products’, what are they actually? And what are the legal grounds for applying the FCTC rules to them? Although it would be going too far to conclude that the decision locks the Parties into a more permissive trajectory, it preserves the existing conceptual confusion. Overall, considering the practice of many Parties of closely following the recommendations of the COP, the decision looks at least like a missed opportunity to comprehensively address HTPs in the FCTC framework.

Yet, considering the circumstances of the adoption of the decision, this outcome is hardly surprising. As described above, the WHO had not provided any specific recommendation in its report on HTPs. As far as we know, no other stakeholder highlighted the importance of regulating HTP devices. In the policy briefing presented at the COP, the FCA had noted that ‘it is important for Parties to ensure that their existing tobacco control measures cover both elements [tobacco and the electronic devices].’ [51] However, this statement was not included among the main recommendations of the policy briefing. Moreover, the FCA’s recommendation of not rushing into adopting a decision on HTPs (‘silence is golden’) certainly did not help to shift the debate on the regulation of HTP devices. Focused as it was on making sure that the HTPs’ nature as tobacco products was reaffirmed, the main stakeholders (including the WHO and the FCA, but possibly others too) seem to have underestimated the regulatory challenges posed by HTP devices.

### Twice is not a mistake: waterpipes and the recurring tobacco/device dichotomy

Hurry, nonetheless, may be only one of the reasons for the COP’s unsatisfactory decision on HTPs. The COP, in fact, seems to have generally failed to appreciate the importance of ensuring that regulations are applied both with respect to tobacco and with respect to the devices meant to be used for its consumption. Note that HTPs are not the only products that pose the problem of the regulatory tobacco/device dichotomy. Most notably, the challenge has been posed by waterpipes.

Waterpipes (a category that includes a variety of products under different names such as ‘hookah’ or ‘shisha’) are smoking devices where tobacco is inhaled through a hose, after it is burnt and passes through a water vase. Although waterpipes have long been used, especially in the Middle East, they have recently become a global phenomenon that warrants careful consideration [70]. Like HTPs, there is an important regulatory dichotomy between tobacco/devices with respect to waterpipes. The ‘apparatus’ (as it is often called) can be a massive and attractive product, but it is often unregulated, or at least insufficiently regulated [71]. Yet, from a sales’ point of view, the apparatus is for waterpipes what a package is for cigarettes: a marketing tool.

This point can be illustrated by imagining a typical purchase in a waterpipe shop. When a customer enters the shop, the first appealing object (s) he would see is the waterpipe apparatus; which in some cases could be in golden colour or decorated with symbols and animal figures. For a customer, the decision to buy a waterpipe will be based on this first impression, i.e. on the attractive power of an apparatus. It is only in the second phase that a customer would buy the tobacco to be smoked using the apparatus. Accordingly, any retail requirement imposed on the tobacco (e.g. a visual display ban, or health warnings) would have limited effectiveness: at that point, as the decision to purchase the waterpipe will have already been made. It is for this reason that Turkey has been praised for being the only country that has adopted a law that requires health warnings to be placed on waterpipe apparatuses [66]. While this does not count as a full application of FCTC provisions to waterpipe apparatuses, it is certainly an important step in that direction.

The COP has devoted two separate decisions to waterpipes, neither of which is fully satisfactory. In 2014 the COP adopted a first exploratory decision on the issue, inviting Parties to: 1) include data on waterpipes in their tobacco surveillance systems; and 2) ‘strengthen their implementation of the WHO FCTC in relation to waterpipe tobacco products, through the integration of waterpipe prevention and control into tobacco-control measures’. [72] The same decision requested the WHO and FCTC Secretariat to provide more information on waterpipes and on best regulatory practices [73].

Following this request, in 2016 the WHO Secretariat provided a comprehensive report which well highlighted the regulatory challenges posed by waterpipes, and the need to adopt tailored approaches [74]. The report included, inter alia*,* best practices, such as the experience of Turkey with health warnings on waterpipe apparatuses. The response of the COP, however, was mild. The second decision it adopted on waterpipes ‘invite [d] Parties … to consider the full application of the WHO FCTC articles in all aspects of waterpipe use, including tobacco used in the waterpipes and accessories as indicated in [the WHO report]’. [75] This text leaves some room for interpretation of the term ‘accessories’. The WHO report uses the term ‘accessories’ to indicate individual accessories (such as ‘charcoal, filters and mouthpieces’) [76]. Conversely, the WHO report refers to the main apparatus by saying ‘accessories and …. waterpipes themselves’. [65] Is, therefore, the apparatus included in the term ‘accessories’?

Even assuming the COP decision covers the main waterpipe apparatus, and leaving aside the interpretative problems, the substance of the decision is neither satisfactory. Despite having a slightly stronger language than the decision on HTPs (which only recommended the Parties to *consider prioritizing* the application of FCTC to HTP devices *where appropriate*), the COP’s decision on waterpipes does not employ any binding or at least declaratory language (i.e. by simply identifying the scope of the relevant provision without however creating the impression that it imposes any additional obligation on State-Parties [77]).

While the reference to the WHO report provides some guidance to the countries that may wish to adopt more comprehensive regulations, the COP decision still misses the most important point: to clearly state that, for products such as waterpipes, there should be no dichotomy between tobacco/device, as the two components ought to be considered as one integrated product. The decision on waterpipes, therefore, seems to confirm that the decision on HTPs is not only the result of rushed negotiations, but of a repeated narrow approach to regulating tobacco products. Rephrasing a famous quote, the COP keeps concentrating on the finger (tobacco), and does not look at the moon (the bigger picture of how tobacco products are marketed and used).

One may ask what are the reasons behind such a constrained approach of the COP towards various devices used to consume tobacco products in a narrow sense. We can only speculate here, but one possible explanation may be the reluctance of the Parties to stretch the language of the Convention too much, and as a consequence be accused of overstepping the COP’s mandate (i.e. amending the Convention without following the required procedures). The COP’s decisions are also taken by unelected government officials, while any amendment of the Convention would require an involvement of (elected) national parliaments, giving it necessary legitimacy. Moreover, although the COP’s decisions are taken by a majority vote, they apply to all Parties. In the case of a treaty amendment only those Parties that accepted the change are bound by the modification. Thus, adopting an interpretation which is too broad, may undermine one of the basic principles of public international law that any obligations arise from a consent of States (or other subjects).

However, as argued in this article there seems to be enough flexibility in the language of the Convention to properly address the problem of devices, without being accused of trespassing the limits imposed by the Convention on its COP.

### Towards a teleological interpretation of the term ‘tobacco products’

It is our view that a literal reading of the text of the FCTC already allows for the consideration of such products as integrated products. Indeed, the definition of tobacco products not only includes products that are ‘partly’ made of tobacco, but also includes their functional characteristics: ‘ … which are manufactured to be used for smoking, sucking, chewing or snuffing’ (Art. 1(f)). The functional characterisation of tobacco products underlines the importance of not regulating individual parts separately. What matters is not the percentage of raw tobacco that can be found in a product, but the product as a whole, by reason of its function.

A teleological reading of the same definition (which highlights the ‘context’ and ‘purpose’ of the treaty), only reinforces this interpretation. If the objective of the FCTC is ‘to protect present and future generations from the devastating health, social, environmental and economic consequences of *tobacco consumption* and exposure to tobacco smoke’ (Art. 3, emphasis added), and if this effort is frustrated by a lack of comprehensive regulation of HTPs or waterpipes, then the term ‘tobacco products’ cannot but be interpreted as comprising all the components of such products.

Since in any case doubts seem to persist, the COP could take the initiative to provide a clarification of the definition. It is fully within the powers of the COP, for example, to adopt an amendment to the FCTC following the procedure established in Art. 28. This procedure, however, has never been used and appears cumbersome, for it requires the submission of a formal instrument of acceptance of the amendment by at least two thirds of the Parties. In this context, one should also take into account the political economy behind amending any provision of the Convention. Such a decision is always risky business as it opens the possibility of watering down specific obligations of the FCTC. The long history of the tobacco industry’s undue influence in the WHO and FCTC activities has made the Parties very wary of potential attempts to weaken FCTC obligations and recommendations [78].

Another less risky route could be more easily pursued. For matters that would not entail changes to the treaty (such as the clarification of a definition), the COP could simply adopt a relevant decision. Although the FCTC text does not provide for such a ‘simplified amendment procedure’, any operator interpreting the treaty would be required to take into account ‘any subsequent agreement between the parties regarding the interpretation of the treaty or the application of its provisions’ as required by Art. 31.3 of the VCLT. In our opinion, and in line with the position of the International Law Commission [79], FCTC COP’s decisions can be regarded as such subsequent simplified agreements that have an impact on interpretation of specific terms used in the FCTC. This is true even if the FCTC does not provide the COP with the right to issue authoritative interpretations of the Convention’s provisions.

In either case, should the COP decide to clarify the meaning of tobacco products, our proposal would be to state that: ‘The definition of “tobacco products” provided in Art. 1(f) of the FCTC is to be understood as including the devices specifically designed to be used for smoking or consuming tobacco in any form.’

### The problem of the FCTC’s suitability for HTPs

Leaving aside the issue of the FCTC’s applicability, a separate but related problem is the suitability of its disciplines for the regulation of HTPs. Note that this is a normative question that relates not so much to the actual applicability of the Convention and its extent, but rather to the desirability of such an approach.

We believe, for the reasons explained above, that treating HTPs, at least as of now, as traditional tobacco products is the correct approach. Although the science behind HTPs is still at an initial stage of development, it is already clear that those products, being based on tobacco, do pose certain health risks, while their emissions contain nicotine (which is highly addictive) as well as other hazardous chemical substances. They may be indeed safer than their combustible counterparts, but it is too early to determine with sufficient precision the extent of risks connected with their use, or the broader societal implications of this technology. Consequently, until more scientific evidence is available, which would allow for a fuller assessment of the risks, treating HTPs as tobacco products appears to be a rational middle-way strategy. On one hand, under such an approach HTPs can be still marketed (similarly as their traditional counterparts), thus preventing regulatory overreach (i.e. the complete elimination of products that may turn out to be less risky to their users than combustible tobacco). Although on the basis of Art. 2 FCTC Parties can always go above and beyond the Convention’s obligations (e.g. by introducing complete bans for those products), as noted above, in practice most of them see the Convention as the main point of reference that determines their domestic regulatory frameworks. So frequently the minimum standard becomes the actual standard. On the other hand, HTPs remain subject to all existing tobacco control restrictions (e.g. advertising bans, age limits, health warnings, and the protection of public health policies from commercial and other vested interests of the tobacco industry). This, in turn, minimize the various (actual or potential) risks indicated above, provided of course that the same restrictions are applied to the HTP devices and tobacco sticks.

Having said the above, the regulatory treatment afforded to HTPs may be modified in the future once sufficient scientific evidence emerges. We can see at least three possible scenarios. One, if *HTPs are determined as not significantly reducing health risks for their individual users (ex-smokers)*, we would recommend consideration of a total sales ban rather than the application of a conventional tobacco control regime, e.g. one that is provided by the FCTC. If those products are not less harmful, there is little reason to keep them on the market (note that HTPs were introduced by the tobacco industry as a potentially safer alternative). Such an approach would have some additional advantages. First, it would limit the variety of the products available on the market and as a consequence decrease the overall attractiveness of tobacco products for consumers. Second, it could also reduce the risk for consumers of being misled, performing the same function as the single presentation requirement adopted by Uruguay [80] or a ban on deceptive descriptors on packaging (such a ‘light’ or ‘ultra-light’), which is already endorsed by the FCTC.

Under the second scenario, HTPs are *found to reduce individual health risks, but at the same time some or all potential systemic risks turn out to be real and difficult to regulate* (e.g. through targeted regulation). Of course, such a situation will require an in-depth and comprehensive cost-benefit analysis. However, as an initial proposition we would suggest maintaining the current classification of HTPs as tobacco products, and consequently applying the traditional tobacco control regime to them (both to tobacco sticks and devices). As noted above, such an approach would still allow the current smokers to switch to less risky products, but at the same time would not undermine the existing regulations aimed at the eradication of tobacco consumption. This should minimize the potential impact of HTPs on the re-normalization of smoking, prevent tobacco companies from exercising influence on public health regulatory processes, and eliminate (or at least limit) the gateway effect (if any).

In the last scenario, *HTPs are determined to reduce individual health risks and not to pose any significant systemic risks if properly regulated*. Since, as explained above, these products are both addictive and harmful, leaving them completely unregulated is not an advisable option. Rather, the FCTC Parties will need to develop a *sui generis* regulatory model that will attempt to maximize the benefits resulting from switching from traditional tobacco products to HTPs, but at the same time address systemic concerns and minimize other risks. This could, for example, mean keeping the age and advertising restrictions but allowing for certain types of targeted commercial communications relating to the relative risks posed by these products. The Parties may also consider different tax treatment of traditional tobacco products and HTPs. It should be noted that the early regulatory models of this kind are already used by some states. For example, the European Union designed such a model, which although it is heavily based on the traditional tobacco control regime, also expressed its own particularities that sometimes go beyond the conventional requirements (e.g. notification obligations) but with respect to other aspects are apparently less strict (e.g. different health warnings) [81]. A similar system for so-called modified risk tobacco products (MRTP) also exists in the United States. The first authorizations under the MRTP pathway has been already granted by the FDA to Swedish Match USA, Inc. for eight snus smokeless tobacco products [82]. More recently the FDA has also authorized marketing of IQOS as MRTP, with reduced exposure information [83].

Some of the above changes may require formal amendment of the FCTC text (with all the difficulties indicated above). While the approach advocated under the second scenario calls for the current status quo (provided that all components of HTPs are covered), and may be implemented through a correctly formulated COP decision, the two other approaches go beyond the FCTC requirements for tobacco products. Therefore, a simple decision of the COP may be insufficient as the proposed modifications can hardly be dealt with in the process of interpretation [84]. This, however, does not mean that the COP as such can do nothing. It is always possible for the COP to recommend, in a non-binding manner, a specific policy related to the implementation of the FCTC objectives. To this end, the COP can use, for example, Art. 5.2 of the Convention which calls the Parties for development of ‘appropriate policies for preventing and reducing tobacco consumption, *nicotine addiction* and exposure to tobacco smoke (emphasis added).’

Note also that it is not only science which will determine the future shape of the regulatory model for HTPs under the FCTC. One of the factors that also seems to be highly relevant in this context is the conceptualization of the objective of the Convention (or more generally, the foundations of the current tobacco control paradigm). It should be recalled that Art. 3 of the FCTC states that its goal is to ‘protect present and future generations from the devastating health, social, environmental and economic consequences of tobacco consumption and exposure to tobacco smoke …. to reduce continually and substantially the prevalence of tobacco use and exposure to tobacco smoke.’ As noted above, an interpreter of a treaty is required to look not only at the ordinary meaning of the words, but also needs to read the relevant terms in light of its object and purpose of a particular agreement. If one concentrates on such elements as ‘tobacco consumption’ and ‘prevalence of tobacco use’ as elements that determines the purpose of the FCTC, HTPs should be rather banned, or at least regulated as traditional tobacco products irrespectively of what the science says about their risk profile. At the end of day these products may preserve nicotine addiction by allowing the current users of traditional tobacco products to simply switch rather than to quit completely. In other words, HTPs may be based on a false assumption that smokers cannot and will not quit smoking. On the other hand, a different approach may be warranted if ‘protect [ing] present and future generations from the devastating health, social, environmental and economic consequences of tobacco consumption’ is considered as one of the central goals of the FCTC. This language implies the existence of a harm reduction dimension (an issue which remains highly controversial in the context of the Convention) [85], and seems to be more friendly towards the idea of considering HTPs as one of the available solutions for dealing with the tobacco epidemic (of course only after their harm reduction potential is confirmed by rigorous and independent scientific research and in parallel with the other tobacco control measures promoted by the FCTC).

The current picture is mixed at best. On the one hand, there are various tobacco products available in many FCTC Parties that are less harmful – at least at the level of individual users – than cigarettes (e.g. snus). However, these products are not subject to any lesser obligations under the Convention. On the other hand, the COP has decided to take a more liberal approach when dealing with ENDS. In particular, the COP recommended to the Parties, as one of the available options (alongside the prohibition or regulation of ENDS as tobacco or medicinal products), applying the regulatory measures referred to in a report prepared in 2016 by the WHO [86]. This report identifies specific measures that are calibrated to the risks posed by ENDS, such as prevention of the initiation of ENDS by non-smokers and youth; minimization of potential health risks to ENDS users and protection of non-users from exposure to their emissions and the prevention of unproven health claims from being made about ENDS. At the same time, the overall design of these measures seems to be less demanding than the FCTC regime for traditional tobacco products [87]. It remains to be seen which vision of the Convention will prevail in the future.

## Discussion and conclusions

As smoking prevalence has declined in the last decades in the many high-income and middle-income countries, there is a diffused perception that the problem of tobacco consumption, at least as a matter of principle, is solved. This is not the case. The emergence of new tobacco products, such as HTPs, is posing serious challenges for the public health experts, decision-makers and the broader public. This article tries to explain the reasons why regulating these products is so complex, and to discuss how the current and future problems can be technically addressed by the international forum in which the countries design the optimal tobacco control solutions (i.e. the FCTC).

We recognize that the problems posed by HTPs transcend the boundaries of one discipline. At the same time, we see an important added value in the focused legal research that concentrates on the actual legal text. Such an investigation helps to recognize the existing legal limits for policy actions, identify those regulatory aspects that require modifications, map the parameters for the possible regulatory approaches and explain how they can be technically implemented. In this sense, the present research contributes to the understanding of how novel and emerging tobacco products, both at the international and national level, are and should be addressed.

Our analysis clearly shows that HTPs can (and should) be classified as ‘tobacco products’ under the FCTC. They are partly made of the leaf of tobacco and used for smoking. At the same time, we recognize that the Convention remains to some degree ambiguous with respect to its exact scope. In particular, it is not clear whether it only covers sticks or extends its disciplines to HTPs devices as well. Although the COP has tried to deal with this ambiguity, the ultimate wording of the relevant decision remains unsatisfactory. The language used by the COP appears to be overly soft (i.e. ‘consider prioritizing’), too broad (i.e. ‘where appropriate’ which provides only limited guidance as to identification of the relevant FCTC provisions) and conceptually unclear (i.e. the apparent distinction between HTP devices and novel and emerging tobacco products). Consequently, the decision seems to provide inadequate assistance to the Parties, when regulating such products. In our opinion the existing ambiguity can be properly addressed through a COP decision, in which the Parties would express their understanding of the term of ‘tobacco products’. Such a definition should have a functional character and clearly state that it also includes the devices specifically designed to be used for smoking or consuming tobacco in any form. The definition will be relevant not only for HTPs but also for other devices that are used for tobacco consumption (e.g. waterpipes). Although we believe that this can be done without a formal amendment of the FCTC, the Parties may also consider inserting a new text into the Convention.

An amendment to of this kind could be also an opportunity to update the FCTC more generally in light of the recent changes in the market. As this article has shown, the COP has struggled to find a satisfactory regulatory approach not only for HTPs, but also for waterpipes. The recent trend toward the legalization of marijuana is also posing some parallel or overlapping problems to existing tobacco control regulations. It is perhaps time for the FCTC Parties to recognise that the market for tobacco and smoking products has significantly evolved since the adoption of the FCTC, and it is going to be less and less focused on cigarettes - the ailing ‘king’ product. If the Parties want the Convention to remain the central forum in the field, they should consider how to make its text more flexible and how to enable its bodies to respond more rapidly to the emergence of new products. In the absence of a prompt and strong recommendation from the FCTC, States are more likely to take different and possibly divergent regulatory approaches – as it has been the case for ENDS and now for HTPs. In this context perhaps environmental treaties may be considered as an inspiration, as they are designed in a way that allows for a quick response to new developments. This article may be regarded as a call (and a starting point) for further research in this area.

We argue that qualifying HTPs as a ‘tobacco product’ (in a functional sense, as a combination of both the device and sticks) and subjecting them to all FCTC disciplines is, in light of our current scientific knowledge, a rational approach. Such an approach can be, however, modified in the future once a sufficient amount of new evidence on their risk profile is collected. At the same time, any decision on the optimal regulatory model for HTPs should take into account not only individual health risks (and potential benefits, if any), but also the systemic concerns identified in the literature. In this context, we have pinpointed three possible scenarios (i.e. HTPs are determined as not significantly reducing health risks for their individual users (ex-smokers); HTPs are found to reduce individual health risks, but they also create certain systemic risks and HTPs are determined to reduce individual health risks and not to pose any significant systemic risks if properly regulated) and proposed different regulatory approaches for each of the scenarios. Our aim here was modest, as we only intended to identify the starting points for the development of the optimal regulatory models once a new knowledge on risk and benefits of HTPs becomes available. The more precise guidance requires additional research which, we hope, this article will provoke.

Last but not least, the article recognizes the state of scientific research is not the only factor that will determine the fate of HTPs under the Convention (the assumption which is taken for granted by some experts). In this context, we note that conceptualization of the FCTC’s objectives is equally important. If a complete eradication of the tobacco epidemic is the ultimate goal, reduced levels of risk for individual users may not be enough to justify the different (and arguably more lenient) regulatory regime. This in turn leads to the more general question, which largely remain under-investigated, of the values that underlie the current tobacco control paradigm.

## Data Availability

Not applicable.

## Abbreviations

Art.: Article
COP: Conference of the Parties (of the Framework Convention on Tobacco Control)
ENDS: Electronic nicotine delivery systems
EU: European Union
FCA: Framework Convention Alliance
FCTC: Framework Convention on Tobacco Control
HTPs: Heated tobacco products
Para: Paragraph
WHO: World Health Organization
